# Characterization of *agr-*like Loci in *Lactiplantibacillus plantarum* and *L. paraplantarum* and Their Role in Quorum Sensing and Virulence Inhibition of* Staphylococcus aureus*

**DOI:** 10.1007/s12602-025-10476-8

**Published:** 2025-02-19

**Authors:** Weizhe Wang, Ifigeneia Kyrkou, Martin S. Bojer, Dina Kalloubi, Abdul Jabbar Kali, Miguel Alena-Rodriguez, Jørgen J. Leisner, Stephanie Fulaz, Hanne Ingmer

**Affiliations:** 1 Bacterial & Viruses Section, Department of Veterinary and Animal Sciences, University of Copenhagen, Stigbøjlen 4, 1870 Copenhagen, Denmark; 2 Center for Biopharmaceuticals & Department of Drug Design and Pharmacology, Faculty of Health and Medical Sciences, University of Copenhagen, Jagtvej 160, 2100 Copenhagen, Denmark

**Keywords:** Probiotics, Quorum sensing, *agr* system, Anti-virulence therapy, Auto-inducing peptides (AIPs), Cross-species communication

## Abstract

The pathogenicity of *Staphylococcus aureus* is largely regulated by the *agr* quorum sensing (QS) system encoded by *agrBDCA*, which coordinates virulence factor production through secretion and sensing of auto-inducing peptides (AIPs). *agr*-like systems are also present in coagulase-negative staphylococci, and several of these encode AIPs that inhibit *S. aureus* QS. In lactic acid bacteria, a similar locus was previously identified in *Lactiplantibacillus plantarum* WCSF1 termed *lamBDCA*. Here, we characterized the *lamBDCA* locus in *L. plantarum* LMG 13556 and *L. paraplantarum* CIRM-BIA 1870, and explored the effects on *S. aureus* QS. Notably, we found that co-cultivation with *L. paraplantarum* significantly inhibits *S. aureus* QS and hemolysin production, while less so for *L. plantarum*. The inhibition by *L. paraplantarum* was lost upon disruption of its *lamBDCA* locus, suggesting that the *L. paraplantarum* AIP mediates cross-species interference with *S. aureus agr* activation. Transcriptomic analysis revealed that *lamBDCA* in *L. paraplantarum* controls the expression of genes belonging to various functional categories, including stress response and metabolism. The latter includes genes encoding riboflavin (B2 vitamin) biosynthesis, which enabled the growth of the *L. paraplantarum lamB* mutant in the presence of roseoflavin, a toxic riboflavin analogue. Collectively, our results show that *L. paraplantarum* CIRM-BIA 1870 interferes with *S. aureus* virulence gene expression through QS suppression, and they implicate QS in the probiotic properties of *L. paraplantarum*.

**Supplementary Information:**

The online version contains supplementary material available at 10.1007/s12602-025-10476-8.

## Introduction

Lactic acid bacteria are Gram-positive bacteria widely used in fermented food production and applied as probiotics, with many species associated with numerous human health benefits. A large group of these bacteria consist of *Lactobacillus* sensu lato including species like *Lactiplantibacillus plantarum* and *L. paraplantarum*, which are known to enhance both innate and adaptive immune responses, as well as protect against infections [1]. They contribute to gut health by competing with pathogenic bacteria for adhesion sites on the intestinal mucosa, thereby preventing pathogen colonization [2]. Additionally, Lactobacilli produce various bioactive compounds with antimicrobial activity [2, 3] and suppress toxin production in pathogens, contributing to their anti-infective properties [4].

*Staphylococcus aureus* is a pathogen for which toxin production is crucial to its virulence. It is an opportunistic bacterium that colonizes about one-third of the human population, primarily residing in the skin, nasal cavities, and gut [5, 6]. It can cause a range of infections, from mild skin conditions to severe bacteraemia [7]. One of the major challenges in treating *S*. *aureus* infections is the increased frequency of variants resistant to antibiotics, including β-lactam resistance in methicillin-resistant *S*. *aureus* (MRSA), decreased vancomycin susceptibility (VISA), and vancomycin-resistance (VRSA). In addition to the increasing lack of usable antibiotics, a prophylactic approach is not possible as no effective vaccines are currently available [8–10]. Therefore, alternative strategies, such as “anti-virulence” therapy, are being explored. This approach aims to reduce pathogen virulence rather than killing the bacteria directly, allowing the host immune system to eradicate the pathogen [11].

A promising target for anti-virulence therapy is the *S. aureus* accessory gene regulator (*agr*) quorum sensing (QS) system, which controls the expression of a wide array of virulence factors. The *agr* system responds to auto-inducing peptides (AIPs) that are secreted by the bacterium and bind to the sensor histidine kinase, AgrC, leading to phosphorylation of the transcriptional regulator AgrA [12]. In stationary growth phase, AIP accumulation induces the *agr* system and triggers the expression of RNAIII, a central regulatory molecule, via the P3 promoter, leading to toxin production, such as α-hemolysin, while repressing adhesin and other surface-associated virulence factors [13, 14].

The quorum molecule of *agr*, the AIP, is a cyclic peptide consisting of a carboxy-terminal 5 amino-acids thiolactone ring and an exotail of variable length [15]. *S. aureus* strains are divided into four *agr* subgroups, each with unique AIPs and corresponding AgrC variations, resulting in a specificity of *agr* activation or cross-inhibition between strains. Similarly, *S. aureus agr* is inhibited by AIP variants produced by other staphylococcal species, such as *S. schleiferi*, *S. warneri*, *S. intermedius*, and *S. delphini* [16, 17], as well as by other cyclic non-AIP peptides [4, 18–20]. Notably, fengycin, produced by *Bacillus subtilis*, significantly reduces *S. aureus* colonization of the gut and nasal passages via repression of the *agr* system [21, 22]. Furthermore, cyclodepsipeptides (e.g., solonamide) from *Photobacterium halotolerans* and dipeptides from *Limosilactobacillus reuteri* have shown inhibitory effects on *S. aureus agr*-mediated QS [4, 23]. These findings suggest that QS interactions within the microbiome influence *S. aureus* prevalence and that probiotics with such capabilities may be a promising way to reduce *S. aureus* colonization.

Homologous *agr*-like systems have also been identified in other bacterial pathogens, including *Listeria monocytogenes* [24], *Enterococcus faecalis* [25], and *Clostridium perfringens* [26], as well as in commensal bacteria, such as *L. plantarum* [27] and *Roseburia inulinivorans* [28]. In *L. plantarum*, the *agr*-like system, known as *lamBDCA*, was first characterized in strain WCFS1 [29]. Similar to *S. aureus,* expression of the LamC histidine kinase and LamA response regulator is induced during stationary growth phase, along with the production of the LamD558 peptide (CVGIW), a cyclic thiolactone peptide that likely results from LamB-dependent processing of LamD [29]. Additionally, *L. plantarum* encodes another two-component system, LamKR, which appears to work in conjunction with LamCA to respond to the LamD558 peptide, influencing cell adhesion and morphology [29, 30]. The structure of AIPs produced by some of these organisms differs from staphylococcal AIPs. While staphylococcal AIPs are cyclic thiolactones stabilized by peptide tails, the AIPs from *L. monocytogenes*, *C. perfringens*, and *L. plantarum* are tailless thiolactones that undergo an S → N acyl shift at neutral pH, converting them into homodetic cyclopeptides [17]. These structural differences can influence the stability and activity of AIPs, potentially impacting their ability to inhibit *agr*.

Here, we investigated the ability of the *lamBDCA*-encoded systems in *L. plantarum* LMG 13556 and *L. paraplantarum* CIRM-BIA 1870 to inhibit *S. aureus agr* quorum sensing. These strains were selected as they encode *agr*-like loci homologous to the well-characterized *lamBDCA* system in *L. plantarum* WCFS1, thus representing an opportunity to expand our understanding of the diversity and functionality of *agr*-like loci in lactiplantibacilli. Strain LMG 13556 was isolated from vegetable ensilage, while strain CIRM-BIA 1870 was isolated from birch sap. Notably, we found that *L. paraplantarum*, but not *L. plantarum*, inhibited the *agr* system and suppressed toxin production in *S. aureus*. This inhibitory effect was mitigated by the inactivation of the *lamBDCA* locus. Additionally, global gene expression analysis of *L. paraplantarum* wild-type and *lamBDCA* mutant strains revealed that this system likely is autoregulated and influences the expression of genes involved in stress response, metabolism, and regulatory pathways. These findings demonstrate that *agr*-like systems in *Lactiplantibacillus* facilitate cross-species communication with staphylococci. Understanding these interactions may pave the way for the development of probiotic-based therapies targeting *S. aureus* infections, offering an alternative strategy to combat antibiotic-resistant pathogens.

## Materials and Methods

### Bacterial Strains and Growth Conditions

*L. plantarum* LMG 13556 and *L. paraplantarum* CIRM-BIA 1870 were grown in all-purpose Tween (APT) media (BD Difco) or De Man-Rogosa-Sharpe (MRS) media (Oxoid) at 30°C, under low oxygen conditions. *S. aureus* strains were cultured in tryptone soya broth (TSB) medium (Oxoid), at 37°C with shaking at 200 rpm. Mix-media, a 1:5 mixture of APT medium and TSB medium, was used as the control for culturing the YFP reporter strains. Media with varying pH was prepared by adjusting the pH of TSB medium with HCl and NaOH aqueous solutions as necessary, with the help of a pH meter (Metrohm). Media was then sterile-filtered with a 0.22-μm syringe filter (Minisart, Sartorius). Where required, antibiotics (Sigma-Aldrich) were added at the following final concentrations: 100 μg/mL ampicillin for *E. coli*; 10 μg/mL chloramphenicol for *L. plantarum* LMG 13556 and *L. paraplantarum* CIRM-BIA 1870 mutants and *S. aureus* reporter strains. All strains used in this study are listed in Table 1.

**Table 1:** Bacterial strains and plasmids used in this study

Strains or plasmids	Species	Relevant phenotype or genotype	Reference or source
Strains			
WCFS1	*Lactiplantibacillus plantarum*		[29]
CIRM-BIA 1870	*Lactiplantibacillus paraplantarum*	Birch sap isolate	Centre International de Ressources Microbiennes
WW101	*Lactiplantibacillus paraplantarum*	*L. paraplantarum* CIRM-BIA 1870 mutant with *lamB* inactivated by single-crossover homologous recombination, Cm^r^	This study
LMG 13556	*Lactiplantibacillus plantarum*	Ensilage of vegetable matter	[31]
WW102	*Lactiplantibacillus plantarum*	*L. plantarum* LMG 13556 mutant with *lamB* inactivated by single-crossover homologous recombination, Cm^r^	This study
AH1677	*Staphylococcus aureus*	*S. aureus agr I* LAC [P3-*yfp*], Cm^r^	[32]
AH1747	*Staphylococcus aureus*	*S. aureus agr III* MW2 [P3-*yfp*], Cm^r^	[32]
AH430	*Staphylococcus aureus*	*S. aureus agrII* 502a [P3-*yfp*], Cm^r^	[32]
AH1872	*Staphylococcus aureus*	*S. aureus agrIV* MNTG [P3-*yfp*], Cm^r^	[32]
8325–4	*Staphylococcus aureus*	*agrI*	[33]
DH10B	*Escherichia coli*	F^−^ mcrA Δ(*mrr*-*hsd*RMS-*mcr*BC) Φ80d*lac*ZΔM15 Δ*lac*X74 *end*A1 *rec*A1 *deo*R Δ(*ara*,*leu*)7697 *ara*D139 *gal*U *gal*K *nup*G *rps*L	[34]
Plasmids			
pUC18-cat		pUC18 derivative with *cat* gene, Cm^r^	This study
pUC18-cat-CIRM-BIA 1870 *lamB*		pUC18-cat derivative with 547-bp *lamB* fragment of CIRM-BIA 1870	This study
pUC18-cat-LMG13556 *lamB*		pUC18-cat derivative with 556-bp *lamB* fragment of LMG13556	This study

### Whole-Genome Sequencing, Genome Assemblies, and Annotations

The DNA of *L. plantarum* LMG 13556 and *L. paraplantarum* CIRM-BIA 1870 was extracted using the DNeasy Blood & Tissue Kit (Qiagen) according to the kit’s handbook. Whole-genome libraries were prepared using the Nextera DNA Flex Library Kit (Illumina) and sequenced either as paired-end 300 bp reads on an Illumina MiSeq (strain 13556) or as paired-end 150 bp reads on an Illumina NovaSeq (strain 1870). Reads below 30 bp and reads below a Phred score of 20 were removed using Cutadapt v4.4 [35], and retained reads were de novo assembled with SPAdes v3.15.3 [36] to an average read depth of 534 × (*L. paraplantarum* CIRM-BIA 1870) and 160 × (*L. plantarum* LMG 13556) as assessed with BBmap v39.01 [37]. Assemblies were submitted to NCBI and annotated with the NCBI Prokaryotic Genome Annotation Pipeline (PGAP) [38] and are available under the RefSeq accession numbers NZ_JBFCVW000000000.1 (*L. paraplantarum* CIRM-BIA 1870) and NZ_JBFCVX000000000.1 (*L. plantarum* LMG 13556). A record of *L. paraplantarum* CIRM-BIA 1870 whole-genome sequence already existed in the NCBI database (RefSeq: NZ_RIOB00000000.1); however, its genome was re-sequenced here for validation purposes.

### Amino Acid Sequence Comparison

The amino acid sequences of the AgrBDCA proteins from *S. aureus* subsp. *aureus* DSM 20231 (RefSeq: NZ_CP104478.1), as well as the LamBDCA and LamKR proteins from *L. plantarum* WCFS1 (RefSeq: NC_004567.2), were obtained from NCBI (Table S1). We selected *S. aureus* subsp. *aureus* DSM 20231 Agr proteins for comparisons because this strain is the type strain of *S. aureus*. The LamBDCA proteins were not labelled as such (see Table S1 for NCBI record annotations), but their identification was aided by their descriptive annotations and topology. The LamK protein of WCFS1 was identified by using LamC of strain WCFS1 as a template in a PSI-BLAST search against *Lactiplantibacillus plantarum* WCFS1 (taxid:220,668). The search was conducted with default settings and a PSI-BLAST threshold of 0.01 with the LamK protein sorted among the generated results according to the identity reported by [30]. The LamR protein was detected in the same manner and by using LamA of WCFS1 as a template. Similarly, potential *lamBDCA* and *lamKR* loci in the genomes of *L. plantarum* LMG 13556 and *L. paraplantarum* CIRM-BIA 1870 were identified with reference to the generated PGAP annotations (see above section) and their topological features. Homologies were confirmed by conducting pairwise amino acid sequence comparisons with the alternate protocol of the pairwise mode of HHpred and default parameter values [39]. For the comparisons, the Lam proteins of *L. paraplantarum* CIRM-BIA 1870 and *L. plantarum* LMG 13556 were used as query sequences and the proteins encoded by the *agrI* system of *S. aureus* subsp. *aureus* DSM 20231 were used as subject sequences. The Lam proteins of strain *L. plantarum* WCFS1 were also included as a reference. Only homologous amino acid sequences were compared and NCBI SeqIDs of the compared amino acid sequences can be found in Table S1.

### *L.plantarum* LMG 13556 and *L.paraplantarum* CIRM-BIA 1870 *lamB* Inactivation Mutants

The procedure for obtaining *lamB* inactivation mutants was the same for *L. paraplantarum* CIRM-BIA 1870 and *L. plantarum* LMG 13556. Chromosomal DNA was isolated using the DNeasy Blood & Tissue Kits, and plasmid DNA was extracted with the GeneJET Plasmid Miniprep Kit. Primers (Table 2) were designed using Geneious Prime (https://www.geneious.com) and synthesized by Eurofins Genomics. Initially, the Gram-negative high-copy vector pUC18 was equipped with the *cat* gene from pIMAY [40] by PCR amplification with primers IMAY-cat-F-BamHI/IMAY-cat-R-SalI, which allows selection for chloramphenicol resistance in Gram-positive species when chromosomally integrated as a single copy, generating pUC18-cat. Subsequently, truncated *lamB* amplicons from the two strains were PCR amplified and cloned into pUC18-cat. These procedures were performed in *E. coli* DH10B by standard heat shock transformation. Resulting plasmids were purified and transformed into the two recipient strains via electroporation, following previously described procedures [41]. Briefly, electro-competent cells of recipient strains were generated by culturing in MRS medium, supplemented with 1% glycine and 0.3 M sucrose, and prepared in 30% polyethylene glycol-1500. Transformation was performed by electroporation at 1.8 kV, 25 µF, and 200 Ω. Recombinants were selected on chloramphenicol MRS agar plates and incubated at 30°C for 48–72 h. Integration of the non-replicating pUC18-cat *lamB* plasmid into the *lamB* gene resulted in *lamB* mutant strains, which was confirmed by PCR.

**Table 2:** Sequence of primers for the *L. plantarum* LMG 13556 and *L. paraplantarum* CIRM-BIA 1870 *lamB* mutants

Primer name	Sequence
IMAY-cat-F-BamHI	5′-GATACAGGATCCATCCCATTATGCTTTGGCAG-3′
IMAY-cat-R-SalI	5′-GATACAGTCGACTTATAAAAGCCAGTCATTAGGC-3′
LMG 13556 *lamB*—KpnI	5′-TACA GGTACC GAAAAGCCAGAACAAAAACTATTG-3′
LMG 13556 *lamB*—EcoRI	5′-TACA GAATTC CTGTTAATGTGAAAACTAATAACAG-3′
CIRM-BIA 1870 *lamB*—KpnI	5′-TACA GGTACC CTGTTCAACGCACTTCAAAATAATC-3′
CIRM-BIA 1870 *lamB*—EcoRI	5′-TACA GAATTC GTTCCTTCATTAGTCTTCCCTCC-3′

### Assessment of *agr* Inhibition

Fluorescent *S. aureus* reporter strains representing *agr* types I-IV (P3-*yfp*) were used to assess the inhibitory effects of *L. plantarum* LMG 13556 and *L. paraplantarum* CIRM-BIA 1870 cell-free supernatants or stationary phase cultures of wild-type and *lamB* mutants. Reporter strains were inoculated from freezer stocks to TSA plates containing chloramphenicol, and subsequently single colonies were inoculated in TSB to an OD_600_ of approximately 0.02, without antibiotics. Overnight cultures of *L. plantarum* and *L. paraplantarum* wild-type and *lamB* mutants were grown in APT medium at 30°C. Cell-free supernatants were obtained by centrifugation at 14,000 rpm for 5 min at room temperature, followed by filtration through a 0.22-μm syringe filter (Minisart, Sartorius). Twenty five microliters of the supernatant was mixed with 75 µL of the *S. aureus* reporter strain in a 96-well black plate (Fisherbrand™), with TSB added to reach a final volume of 150 µL. For co-culture tests, 25 µL of the lactiplantibacilli overnight culture was added to the *S. aureus* reporter strains in a 1:30 (*S. aureus*: *L. plantarum* or *L. paraplantarum*) ratio based on OD_600_. Higher initial OD of *Lactiplantibacillus* was used, because of the slower growth of these strains in an oxygen-rich, TSB-based culture when co-cultured with staphylococci, as seen in growth curves in Figures S1 and S2. Control *S. aureus* cultures were grown in mix-media at the same volume ratio. P3 promoter activity was monitored as accumulated YFP fluorescence (*λ*_exc_ 500 nm, *λ*_*e*m_ 541 nm) using a Synergy H1 Microplate Reader (BioTek) with a continuous 200-rpm orbital shaking, with readings taken every 30 min at 37°C for 24 h.

### Chemical Synthesis of Dipeptides (Diketopiperazines)

For preparing cyclo(L-Phe-L-Pro) and cyclo(L-Tyr-L-Pro), *N,N*-diisopropylethylamine (3.0 mmol), *N*-Boc-L-amino acid (1.66 mmol), and hexafluorophosphate benzotriazole tetramethyl uronium (HBTU, 1.81 mmol) were added to a solution of L-proline methylester hydrochloride (1.5 mmol) in *N,N*-dimethylformamide (DMF, 10 mL), and the mixture was stirred overnight at room temperature. After removal of the solvent under reduced pressure, the residue was dissolved in ethyl acetate (50 mL), washed with saturated aqueous sodium bicarbonate (3 × 20 mL), and brine (1 × 20 mL). The organic layer was then dried over magnesium sulfate, filtered, and concentrated in vacuo. The crude product was purified by flash chromatography (silica gel, heptane/ethyl acetate 1:1) to yield the linear dipeptide, which was suspended in water (50 mL) and stirred at 100°C for 16 h to induce the cyclization. Then, the solvent was removed under reduced pressure and the residue was purified by preparative high-performance liquid chromatography (HPLC) to yield the desired diketopiperazines (DKPs, Table S2).

Cyclo(L-Phe-L-Pro): 190 mg (46% overall yield). ^1^H NMR (600 MHz, DMSO-*d*_6_): δ 7.97 (s, 1H), 7.27–7.19 (m, 5H, H Ar), 4.35 (t, *J* = 5.2 Hz, 1H), 4.08–4.05 (m, 1H), 3.40 (dt, *J* = 11.7, 8.1 Hz, 1H), 3.27 (dt, *J* = 12.1, 6.4 Hz, 1H), 3.08 (dd, *J* = 14.2, 5.0 Hz, 1H), 3.03 (dd, *J* = 14.2, 5.2 Hz, 1H), 2.03–1.98 (m, 1H), 1.73 (tdd, *J* = 9.4, 7.3, 5.6 Hz, 2H), 1.44–1.38 (m, 1H). ^13^C NMR (151 MHz, DMSO-*d*_6_) δ 169.5, 169.1, 137.3, 129.8, 128.0, 126.4, 58.4, 55.8, 44.6, 35.4, 27.8, 21.9. HRMS: *m/z* calcd. for C_14_H_17_O_2_N_2_^+^ [M + H]^+^, 245.1285; found 245.1280.

Cyclo(L-Tyr-L-Pro): 21 mg (49% overall yield). ^1^H NMR (600 MHz, DMSO-*d*_6_): 1H NMR (600 MHz, DMSO-*d*_6_): δ 7.83 (s, 1H), 7.04 (d, *J* = 8.5 Hz, 2H), 6.63 (d, *J* = 8.5 Hz, 2H), 4.24 (t, *J* = 5.1 Hz, 1H), 4.04 (dd, *J* = 10.0, 6.7 Hz 1H), 3.40 (dt, *J* = 11.7, 8.1 Hz, 1H), 3.25 (dt, *J* = 12.1, 6.4 Hz, 1H), 2.95–2.89 (m, 2H), 2.00 (ddt, *J* = 12.0, 6.8, 4.8 Hz, 1H), 1.73–1.70 (m, 2H), 1.43–1.36 (m, 1H). ^13^C NMR (151 MHz, DMSO-*d*_6_) δ 168.9, 165.1, 155.9, 130.8, 127.1, 114.8, 58.4, 56.0, 44.6, 34.7, 27.9, 21.9. HRMS: *m/z* calcd. for C_14_H_17_O_3_N_2_^+^ [M + H]^+^, 261.234; found 261.1225.

### Effect of Dipeptides on *S.aureusagr* Groups

Dipeptide (5 µM, DMSO stock solutions) was added to a 150-µL *S. aureus* reporter culture at OD_600_ of 0.01 in a 96-well black plate (Fisherbrand™). Controls included *S. aureus* grown with an equivalent volume of DMSO. The P3 promoter activity was monitored as accumulated YFP fluorescence (*λ*_exc_ 500 nm, *λ*_em_ 541 nm) using a Synergy H1 Microplate Reader (BioTek) with a continuous 200-rpm orbital shaking, with readings taken every 30 min at 37°C for 24 h.

### RNA Sequencing and Gene Expression Analysis

Wild-type *L. paraplantarum* CIRM-BIA 1870 and the *lamB* mutant were grown in 10 mL APT at 30°C starting from an OD_600_ of 0.01 and harvested at OD_600_ of 1.7 by centrifugation at 6000 × g for 10 min at 4°C. RNA was extracted using the RNeasy Mini Kit (Qiagen). After ribosomal RNA depletion, 150-bp insert strand-specific cDNA libraries were prepared and sequenced by Novogene (UK) using the Illumina NovaSeq platform. Reads were filtered to remove adaptor sequences and omit reads containing N > 10%. Quality control was performed to remove reads where > 50% of bases had a *Q* score of < 5. The final reads were mapped to *L. paraplantarum* CIRM-BIA 1870 (NCBI RefSeq: NZ_RIOB00000000.1) to identify annotated gene transcripts with Bowtie2 v2.3.4.3 [42] and with the mismatch parameter set to two and all other parameters left as default. For novel gene transcript predictions, the RNA-seq reads were assembled according to the reference genome of *L. paraplantarum* CIRM-BIA 1870 using Rockhopper v2.0.3 [43] and StringTie [44], and then compared to known gene structures to predict novel gene transcripts (designated as “Novel” in Results section) within the intergenic regions of the reference genome. The novel transcripts were aligned to sequences in NCBI nr database using Blastx [45] (cutoff: evalue < 1e-5) and annotated using several databases, including GO (Gene Ontology) [46], PFAM (Protein Families) [47], and UniProtKB/Swiss-Prot [48, 49]. Gene expression levels were quantified with FeatureCounts v1.5.0-p3 [50] using an FPKM (fragments per kilobase of transcript per million mapped reads) strategy and an FPKM value of 0.1–1 to distinguish any genes with low expression levels [51]. Differential expression analysis was conducted using DeSeq2DESeq2 v1.20.0 [52] with default settings and the threshold of differentially expressed genes was *p*_adj_ < 0.05. Initial PGAP annotations of the differentially expressed genes were extracted from *L. paraplantarum* CIRM-BIA 1870 (NCBI RefSeq: NZ_RIOB00000000.1) and further curated by reannotating each predicted protein with the HHpred and Blastp databases and default settings [39]. Final annotations were selected only when there was a consensus of annotations from at least two databases used (PGAP, HHpred and Blastp). Results are presented in Table S3. Differentially expressed genes which overlapped with the differentially expressed genes reported by Sturme et al. [29] were identified using Blastp pairwise comparisons.

### Biofilm Assays

A quantitative adherence assay was performed as previously described [30], with minor modifications. Briefly, cells were grown in 2 mL MRS in 24-well plates with or without a glass slide at 30°C for 48 h. The wells were washed twice with water to remove loosely attached cells, and the remaining adherent cells were air-dried for 10 min. To stain the cells, 0.5 mL of 0.1% crystal violet was added, incubated for 30 min, and then washed twice with water. The attached, stained cells were solubilized in 99% ethanol, and absorbance at 595 nm was measured. All assays were performed in triplicate.

### Hemolysin Assay

Hemolysin activity was determined by measuring blood cell lysis as previously described [53]. *S. aureus* 8325–4 was co-cultured with wild-type and *lamB* mutant strains of *L. paraplantarum* CIRM-BIA 1870, while *S. aureus* grown in mixed media served as a control. After 24 h of incubation, cultures were centrifuged at 3000 rpm for 10 min. 200 μL of cell-free supernatant was mixed with 25 μL of red blood cells and 775 μL of PBS buffer and incubated at 37°C for 1 h. Samples were centrifuged at 3000 rpm for 10 min, and hemoglobin released from red blood cells lysis was quantified spectrophotometrically at 450 nm using a Synergy H1 Microplate Reader (BioTek). Controls included 1% Triton X-100 for complete lysis, and *L. paraplantarum* CIRM-BIA 1870 wild-type and *lamB* mutant grown in mix-media for no lysis.

### Roseoflavin Assay

To assess the ability of *L. paraplantarum* CIRM-BIA 1870 mutant and wild-type to grow in the presence of roseoflavin, a tolerance assay was adapted from Burgess et al. 2006 [54]. Overnight cultures of each strain were grown in APT medium supplemented with varying concentrations of roseoflavin (Sigma-Aldrich) ranging from 25 to 400 mg/L at 30°C. The next day, growth was measured by optical density at 600 nm using a Synergy H1 Microplate Reader (BioTek).

## Results

### The *lamBDCA* Operons of *L.paraplantarum* CIRM-BIA 1870 and *L.plantarum* LMG 13556

To assess the *agr*-inhibitory properties of LamBDCA, we obtained the genome sequences of *L. paraplantarum* CIRM-BIA 1870 and *L. plantarum* LMG 13556 by next-generation sequencing. The basic genomic features of the two strains were comparable to that of the type strains within their species [55]. *L. paraplantarum* CIRM-BIA 1870 genome size is 3,228,562 bp and its G/C content is 44.92%, while the type strain DSM 10667 has a genome size of 3.40 Mbp and a G/C content of 43.7%. *L. plantarum* LMG 13556 genome size is 3,368,731 bp and its G/C content is 45.56%, which are comparable to the genome size of 3.45 Mbp and the G/C content of 44.2% for the type strain DSM 20174. Loci homologous to *lamBDCA* from *L. plantarum* WCFS1 [29, 30] were detected utilizing the generated PGAP annotations (Table S1) and the HHpred pairwise amino acid sequence analysis with homologies presented in Table 3. Using the same approach, we detected a region in both strain genomes with homology to *lamKR* from *L. plantarum* WCFS1 (Table S1). Moreover, we detected an 85% and 100% identity of the LamR protein from *L. paraplantarum* CIRM-BIA 1870 and *L. plantarum* LMG 13556 to that from *L. plantarum* WCFS1, and 79% and 100% identity of the LamK protein from *L. paraplantarum* CIRM-BIA 1870 and *L. plantarum* LMG 13556 to that from *L. plantarum* WCFS1.

**Table 3:** Pairwise amino acid sequence identities calculated by HHpred between *S. aureus agr* encoded proteins and those encoded by *lam* loci of *L. plantarum* and *L. paraplantarum*

		*S. aureus*				*L. plantarum* WCFS1			
		AgrB	AgrD	AgrC	AgrA	LamB	LamD	LamC	LamA
*L. paraplantarum* CIRM-BIA 1870	**LamB**	25%	-	-	-	71%	-	-	-
	**LamD**	-	19%	-	-	-	63%	-	-
	**LamC**	-	-	33%	-	-	-	68%	-
	**LamA**	-	-	-	38%	-	-	-	91%
*L. plantarum* LMG 13556	**LamB**	26%	-	-	-	100%	-	-	-
	**LamD**	-	23%	-	-	-	100%	-	-
	**LamC**	-	-	34%	-	-	-	100%	-
	**LamA**	-	-	-	38%	-	-	-	100%
*L. plantarum* WCFS1	**LamB**	26%	-	-	-	100%	-	-	-
	**LamD**	-	23%	-	-	-	100%	-	-
	**LamC**	-	-	34%	-	-	-	100%	-
	**LamA**	-	-	-	38%	-	-	-	100%

### Lactiplantibacilli Mitigate *S.aureus* Quorum Sensing

Given the probiotic potential of lactiplantibacilli, and building on earlier findings of cross-communication between *S. aureus* and other staphylococcal species [16, 56, 57], we assessed their ability to inhibit *S. aureus* quorum sensing. To this end, we employed a set of *S. aureus* reporter strains harboring *agr* types I–IV with P3 promoter-driven yellow fluorescent protein (YFP) expression [32]. Table 4 shows the relative fluorescence level after 24 h in the presence of cell-free supernatants (CFSN) of *L. plantarum* and *L. paraplantarum.* Only minor inhibitory effects, mostly directed towards *agrII*, were observed for CFSN from both strains.

**Table 4:** Modulation of *S. aureus* P3-YFP expression by cell-free supernatants or co-culturing of *L. plantarum* LMG 13556 and *L. paraplantarum* CIRM-BIA 1870

	Strain	*S. aureus* QS activity ^b, c^ (%)			
		*agrI*	*agrII*	*agrIII*	*agrIV*
Cell-free supernatant^a^	*L. plantarum*	127 ± 9%	72 ± 7%	116 ± 8%	92 ± 7%
	*L. paraplantarum*	108 ± 4%	65 ± 6%	94 ± 11%	129 ± 20%
Co-culture^a^	*L. plantarum*	86 ± 6%	59 ± 17%	89 ± 12%	44 ± 12%
	*L. paraplantarum*	33 ± 3%	22 ± 0%	119 ± 13%	40 ± 3%

^a^The relative fluorescence of YFP was measured following exposure to 17% lactiplantibacilli cell-free supernatants or live cells at a 1:30 ratio of *S. aureus* to lactiplantibacilli

^b^24-h endpoint relative expression (YFP fluorescence/OD_600_) value of testing groups were normalized to the corresponding *S. aureus agrI-IV* YFP reporters

^c^Data is represented as mean of three technical replicates from one representative biological replicate

The AIP from *L. plantarum* WCFS1 has been reported to rearrange to a homodetic form at neutral pH [17], which we hypothesize could result in reduced activity. We next tested the effect of live co-cultures. *L. paraplantarum* demonstrated significantly greater inhibition of *S. aureus agr* for *agr* groups I, II, and IV compared to *L. plantarum*, which decreased *agr* activity in groups II and IV, suggesting a stronger interaction in live co-culture conditions (Table 4). Unlike minor effects observed for supernatants, our findings indicate that *L. paraplantarum* considerably affects *S. aureus* quorum sensing when co-cultured, suggesting a context-dependent effect. These data also suggests that the *S. aureus agr* group III is more difficult to inhibit compared to the other *agr* groups. However, our findings may still have therapeutic potential as *agr* group I is the most prevalent one, found in more than 60% of the isolates [58, 59].

### *lamBDCA* in *Lactiplantibacillus* Contributes to *S.aureus* QS Inhibition

**Fig. 1:**
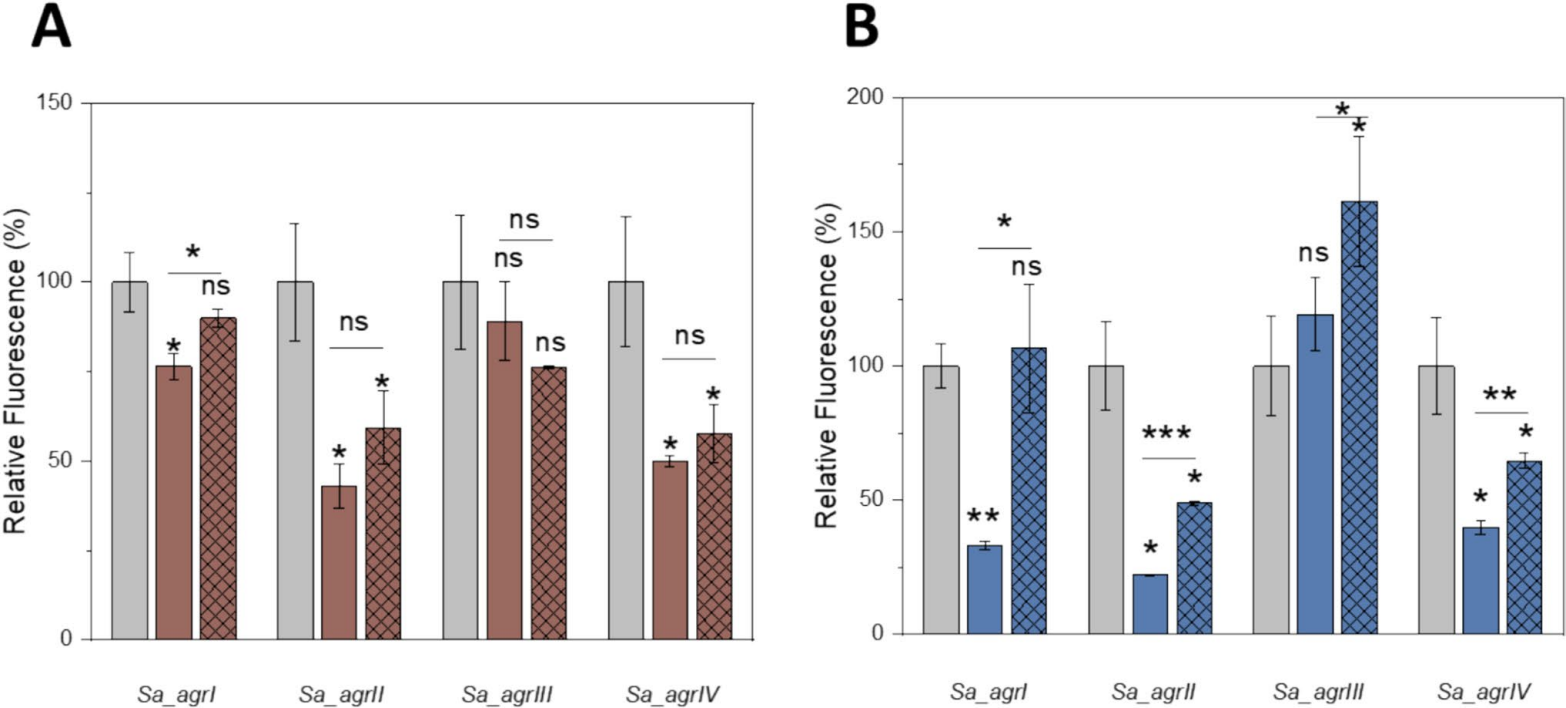
Influence of *L. plantarum* (**A**) *and L. paraplantarum* (**B**) wild-type and *lamB* mutants on *S. aureus* RNAIII expression. Activity of four *S. aureus agr* group reporters assessed by P3-*yfp* (RNAIII) expression measured as fluorescence, either alone or in co-culture with wild-type or *lamB* mutant strains. Relative expression was determined by calculating the ratio of YFP fluorescence to OD_600_, and endpoint values of the 24 h of co-culture groups were normalized to the corresponding *S. aureus agrI-IV* YFP reporters, with these reporters set as the 100% standard for comparison. Data represented as mean ± SD of at least three technical replicates from a representative biological replicate. ns (*p* > 0.05) represents no significant difference between test conditions, * (*p* ≤ 0.05), ** (*p* ≤ 0.01), and ***(*p* ≤ 0.001) represent a significant difference between test conditions. Gray, *S. aureus agrI-IV* YFP reporters; brown, YFP reporters co-culturing with *L. plantarum* LMG 13556; blue, YFP reporters co-culturing with *L. paraplantarum* CIRM-BIA 1870; *lamB* mutants were marked with crosshatch

To determine whether the inhibitory effect of *L. paraplantarum* on *S. aureus agr* is mediated via the *lamBDCA* locus, we constructed *lamB* mutants in both *L. paraplantarum* and *L. plantarum,* in which the *lamB* genes were mutated by insertional inactivation. Growth of the *lamB* mutants was comparable to the wild-type strains (Fig. S1, S2). Importantly, when co-culturing with *S. aureus*, the *L. paraplantarum lamB* mutant showed a significant reduction in quorum sensing inhibition for all *agr* groups. In contrast, the *L. plantarum lamB* mutant exhibited minimal effects, mainly seen in *agr I* (Fig. 1). These results indicate that AIPs produced by *L. paraplantarum* contribute to the inhibition of *S. aureus agr*, while other factors may also play a role. The finding that *L. paraplantarum* but not *L. plantarum lamB* inactivation reduced *agr* inhibition suggests strain-specific differences in *agr*-like quorum sensing that may be linked to unique AIP structures and activities.

### Impact of pH and Dipeptides Produced by *Lactiplantibacillus* on *S.aureus* QS

Considering that additional factors beyond AIPs may interfere with *S. aureus* QS, we investigated the role of pH and the potential effect of dipeptides in our experimental setup. The pH of a 24-h *S. aureus* monoculture in TSB was 6.0, while in a mixed media of TSB and APT it had a pH of 5, *L. paraplantarum* monocultures reached a pH of 4.7. Co-cultures of *S. aureus* with *L. paraplantarum* resulted in a pH of 5.3, and *S. aureus* monoculture mixed with *L. paraplantarum* cell-free supernatants had a pH of 5.6. To directly assess the impact of pH on QS, *S. aureus* was grown in media adjusted to different pH levels (Figure S3). We observed that the *agrIV* system was significantly affected at pH 4.5. However, the pH in mixed cultures did not fall below 5.0 in our experiments, suggesting that pH differences alone cannot account for the observed quorum sensing interference, in accordance with previous studies [60, 61]. These findings support the notion that other factors, such as AIP-mediated cross-inhibition or the production of additional bioactive compounds, are the primary contributors to the inhibitory effects.

**Fig. 2:**
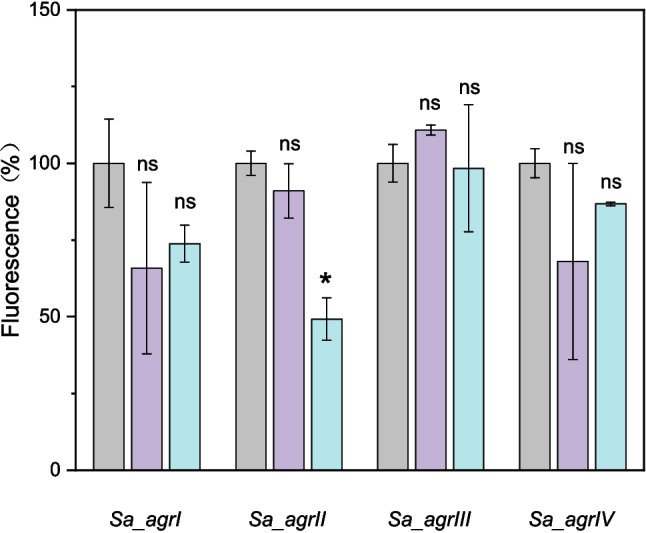
Effect of dipeptides on *S. aureus* RNAIII expression. Activity of four *S. aureus agr* group reporters assessed by P3- *yfp* (RNAIII) expression measured as fluorescence with or without 5 μM dipeptide. Relative expression was determined by calculating the ratio of YFP fluorescence to OD_600_, and endpoint values of the 24-h testing groups were normalized to the corresponding *S. aureus agrI-IV* YFP reporters, with these reporters set as the 100% standard for comparison. Data represented as mean ± SD of at least three technical replicates from a representative biological replicate. ns (*p* > 0.05) represents no significant difference between test conditions, * (*p* ≤ 0.05) represent a significant difference between test conditions. Gray, *S. aureus agrI-IV* YFP reporters; purple, YFP reporters with **DP**1; blue, YFP reporters with **DP2**

Dipeptides produced by *L. reuteri* have previously been shown to influence the production of virulence factors in *S. aureus* [4] and two of these dipeptides, cyclo(L-Phe-L-Pro) (**DP1**) and cyclo(L-Tyr-L-Pro) (**DP2**), have also been identified in *L. plantarum* [62] (Table S2). These dipeptides were therefore synthesized, and their effects on *S. aureus agr* were evaluated. The inhibitory effects of **DP2** on *S. aureus agr-*II (Fig. 2) suggest that the residual inhibition observed in the *L. plantarum lamB* mutant (Fig. 1A) could be partially due to the dipeptide production. Although, these dipeptides have not yet been isolated from *L. paraplantarum*, we hypothesize that the observed inhibition by synthetic dipeptides suggests that multiple mechanisms encoded by lactiplantibacilli such as AIPs and other bioactive molecules, may interfere with *S. aureus* quorum sensing.

### Effect of *L.paraplantarum* on Hemolytic Activity of *S.aureus*

**Fig. 3:**
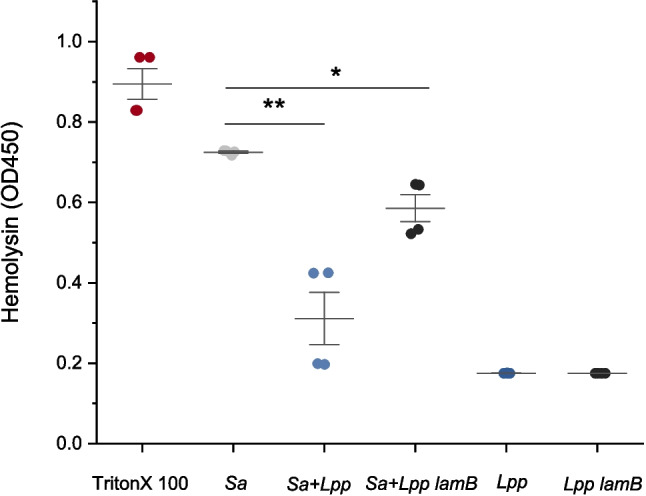
*L. paraplantarum* modulation of *S. aureus* hemolytic activity. Hemolysis was measured using the highly hemolytic strain, *S. aureus* 8325–4 co-cultured with either *L. paraplantarum* wild-type or *lamB* mutant strain. After overnight incubation supernatants were collected, incubated with cow blood cells for 1 h, centrifuged, and absorbance was measured at OD_450_ with 1% Triton X-100 treatment serving as control for full hemolysis. Data represented as mean ± SD of at least three technical replicates from a representative biological replicate. Statistical markers * (*p* < 0.05) and ** (*p* < 0.01) highlight significant differences in activity levels compared to the control

Based on our reporter assays, we anticipated that *agr*-mediated virulence factor production, such as hemolysin in *S. aureus*, would be affected by the presence of *Lactiplantibacillus*. While our reporter assays indicated that *L. plantarum* affected *agrII*- and *agrIV*-mediated virulence gene expression in *S. aureus*, we prioritized *L. paraplantarum* for these studies due to its greater *agr*-inhibitory effects, as observed in the co-culture assays. To examine this, we measured hemolytic activity of *S. aureus* 8325–4, a strong hemolysin producer, after co-culture with wild-type and *lamB* mutant *L. paraplantarum* (Fig. 3). Co-culture with wild-type *L. paraplantarum* resulted in an 80% reduction in hemolytic activity compared to *S. aureus* alone, whereas the *lamB* mutant caused a smaller reduction. This confirms that *agr-*mediated virulence factors, such as hemolysins, are inhibited by the presence of the *lamBDCA* locus in *L. paraplantarum*. The substantial reduction in *S. aureus* hemolysin production by *L. paraplantarum*, and the inability to produce its own hemolysins highlights *L. paraplantarum* potential as a probiotic intervention to suppress virulence factor expression (Figure 3, S4).

### Effect of *lamBDCA* on Biofilm Formation of *L.paraplantarum*

**Fig. 4:**
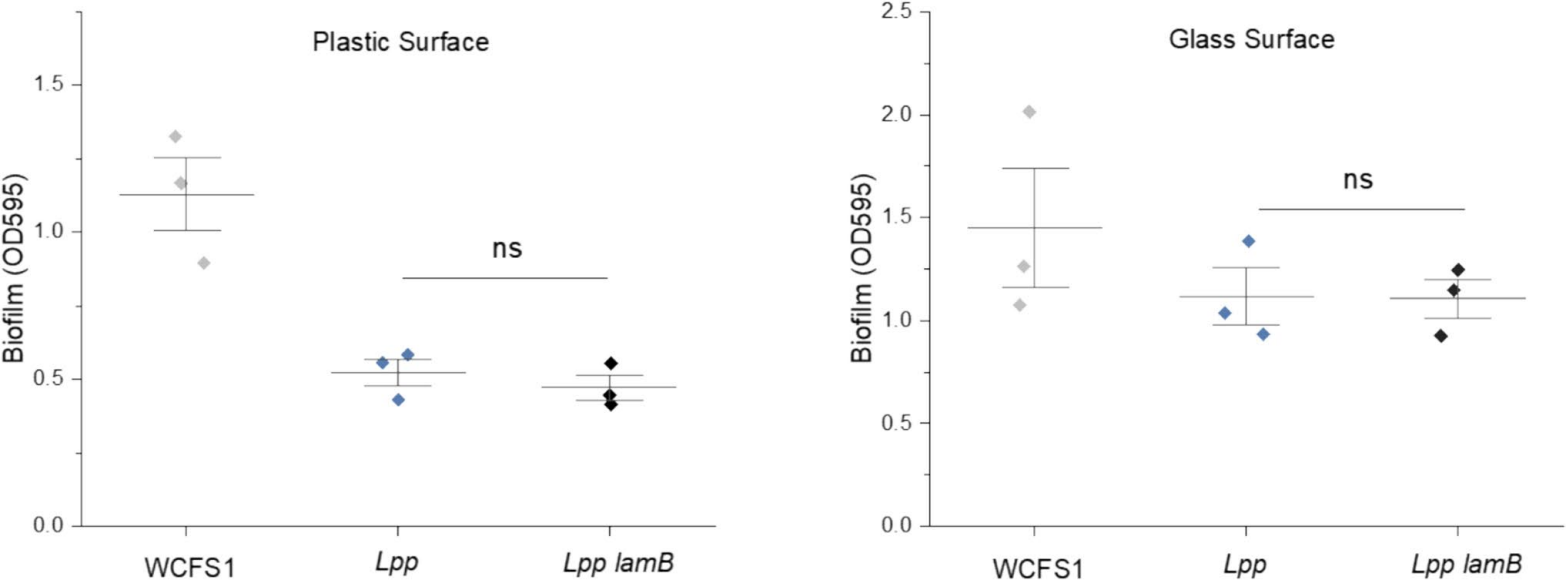
Biofilm formation by *L. plantarum* WCFS1 and *L. paraplantarum* wild-type and *lamB* mutant cells. Bacterial cultures were cultivated in MRS media in the presence of the respective surfaces in 24-well plates. The quantification of the biomass was performed after 48 h using the crystal violet assay, and absorbance was measured at OD_595_. Data represented as mean ± SD of at least three technical replicates from a representative biological replicate. ns (*p* > 0.05) represents no significant difference between test conditions

The *lamBDCA* locus has been previously linked to biofilm formation in *L. plantarum* WCFS1, where its disruption reduced adherence to surfaces and altered cell morphology [29]. To determine whether the *lamBDCA* operon influences biofilm formation in *L. paraplantarum*, we compared the adherence of wild-type and the *lamB* mutant strains on plastic and glass surfaces. Unlike *L. plantarum* WCSF1, the *lamB* mutation did not significantly affect adherence, and both strains formed less biofilm compared to *L. plantarum* WCSF1 on a plastic surface (Fig. 4). This suggests that the *lamBDCA* operon does not universally contribute to biofilm formation across different strains and species. These results indicate that the role of *lamBDCA* in adherence and biofilm formation may be species- or strain-specific, indicating diverse regulatory functions of *agr*-like loci within *Lactiplantibacillus*.

### Effect of *lamBDCA* Operon in *L.paraplantarum* CIRM-BIA 1870 on Global Gene Expression

To examine the impact of the *lam* locus on *L. paraplantarum*, we selected the CIRM-BIA 1870 strain for RNA-seq analysis due to its stronger *agr*-inhibitory activity compared to *L. plantarum* LMG 13556. We compared the transcriptomes of *L. paraplantarum* wild-type and *lamB* mutant strains during the late exponential growth phase using RNA sequencing. The gene expression profile among three independent biological replicates is presented in Figure S5. In total, 658 genes were differentially expressed, with 266 genes upregulated and 392 genes downregulated in the *lamB* mutant compared to the wild-type strain (adjusted *p* value < 0.05, Figure S5).

The *lamBDCA* operon itself (EFP00_RS04540 to EFP00_RS04555) showed a significant reduction in expression in the *lamB* mutant, with fold decreases ranging from − 3.6 to − 5.8. This suggests an auto-regulatory mechanism, where disruption of AIP production breaks the positive feedback loop.

Several other genes showed reduced expression in the *lamB* mutant, including stress response genes (e.g., EFP00_RS04570, EFP00_RS08580, EFP00_RS08585, EFP00_RS08595, EFP00_RS08600, and Novel00154), hematin-dependent catalase (EFP00_RS04565, Novel00074), phage holin (EFP00_RS00340), SDR family oxidoreductase (EFP00_RS01460), KUP/HAK/KT family potassium transporter (EFP00_RS08000), and the ATP-dependent Clp protease subunit ClpL (EFP00_RS04535) (Tables 5, S3 and S4). These changes align with observations from the *L. plantarum* WCFS1 *lamA* mutant [29, 30]. Notably, some stress-related genes, such as universal stress protein (EFP00_RS09265) and alkaline shock protein (EFP00_RS08585) were nearly threefold less expressed in the *lamB* mutant compared to the wild-type. Additionally, two global transcriptional regulator genes, Spx/MgsR family RNA polymerase-binding regulatory proteins (EFP00_RS04560 and EFP00_RS04615), were downregulated − 6.6 and − 3.6-fold, respectively. Interestingly, we also observed reduced expression of prophage-encoding genes (EFP00_RS00340 to EFP00_RS00415) in the *lamB* mutant compared to the wild-type strain. In *Vibrio cholerae* and *V. anguillarum*, it was demonstrated that a host-encoded quorum sensing system controls the decision between lysis and lysogeny of temperate phages [63, 64]. Similar may be the case for the *L. paraplantarum* prophage; however, future studies will be needed to examine this.

**Table 5:** Differentially expressed genes between *L. paraplantarum lamB* mutant and the wild-type strain (|log2fold change|> 2.0)

Gene_ID	Log2fold change	Description
EFP00_RS04540	− 3.6	Homologue of accessory gene regulator protein B, LamB
EFP00_RS04545	− 5.6	Cyclic lactone autoinducer peptide precursor, LamD
EFP00_RS04550	− 5.8	Homologue of histidine kinase sensor protein C, LamC
EFP00_RS04555	− 5.8	Homologue of accessory geneResponse Regulator protein A, LamA
EFP00_RS00340	− 2.3	Phage holin
EFP00_RS00345	− 2.4	Hypothetical phage protein
EFP00_RS00350	− 2.3	phage endolysin
EFP00_RS00355	− 2.1	Hypothetical phage protein
EFP00_RS00360	− 2.3	Hypothetical phage protein
EFP00_RS00365	− 2.2	Phage tail protein
EFP00_RS00370	− 2.1	Phage tail protein
EFP00_RS00375	− 2.1	Phage tail protein
EFP00_RS00390	− 2.1	Phage major tail protein
EFP00_RS00395	− 2.1	Phage tail protein
EFP00_RS00400	− 2.2	Phage tail protein
EFP00_RS00405	− 2.1	Phage structural protein
EFP00_RS00410	− 2.1	Phage head–tail connector protein
EFP00_RS00415	− 2.1	Hypothetical phage protein
EFP00_RS01460	− 2.7	SDR family oxidoreductase
EFP00_RS02095	− 2.5	Hypothetical protein
EFP00_RS02100	− 2.6	Hypothetical protein
EFP00_RS02715	− 2.0	Zinc-binding dehydrogenase
EFP00_RS04220	− 2.3	Pyridoxamine 5′-phosphate oxidase family protein
EFP00_RS04530	− 6.0	Hypothetical protein
EFP00_RS04535	− 3.0	ATP-dependent Clp protease ATP-binding subunit ClpL
EFP00_RS04560	− 6.6	Spx/MgsR family RNA polymerase-binding regulatory protein
EFP00_RS04570	− 2.3	GlsB/YeaQ/YmgE family stress response membrane protein
EFP00_RS04615	− 3.6	Spx/MgsR family RNA polymerase-binding regulatory protein
EFP00_RS08000	− 3.0	KUP/HAK/KT family potassium transporter
EFP00_RS08580	− 3.6	GlsB/YeaQ/YmgE family stress response membrane protein
EFP00_RS08585	− 3.5	Alkaline shock response membrane anchor protein, AmaP
EFP00_RS08590	− 3.2	Hypothetical protein
EFP00_RS08595	− 3.7	Alkaline shock protein 23/Gls24 family envelope stress response protein
EFP00_RS08600	− 3.4	Alkaline shock protein 23/Gls24 family envelope stress response protein
EFP00_RS09265	− 3.2	Universal stress protein
EFP00_RS09415	− 2.7	CsbD family protein
EFP00_RS09420	− 2.4	Integral membrane protein
EFP00_RS09425	− 2.5	Integral membrane protein
EFP00_RS09430	− 2.3	Diacylglycerol kinase family protein
Novel00072	− 3.1	Histidine kinase with LytTr DNA-binding domain
Novel00074	− 4.5	Hematin-dependent catalase
Novel00154	− 2.5	Alkaline shock protein 23
Novel00170	− 4.0	Universal stress protein family
EFP00_RS14825	2.2	tRNA-Asp
EFP00_RS00845	7.6	5-amino-6-(5-phosphoribosylamino) uracil reductase, RibD
EFP00_RS00850	7.7	Riboflavin synthase, RibB
EFP00_RS00855	7.5	Riboflavin biosynthesis protein, RibA
EFP00_RS00860	7.1	6,7-dimethyl-8-ribityllumazine synthase, RibH
EFP00_RS00870	4.4	NAD(P)H-binding protein
EFP00_RS00875	4.2	NAD(P)H-binding protein
EFP00_RS04190	2.2	Hypothetical protein
EFP00_RS07265	2.1	tRNA-Thr
EFP00_RS14140	2.0	Imidazole glycerol phosphate synthase subunit, HisH

Conversely, increased expression of genes related to histidine biosynthesis (operon EFP00_RS14115 to EFP00_RS14165) was observed in the *lamB* mutant. Furthermore, the most significantly increased expression levels were detected in the riboflavin biosynthesis operon (EFP00_RS00845 to EFP00_RS00860), with fold changes of 7.6-, 7.7-, 7.4-, and 7.1-, respectively. This strongly indicates that the *lamB* mutant may be overproducing riboflavin (vitamin B2).

**Fig. 5:**
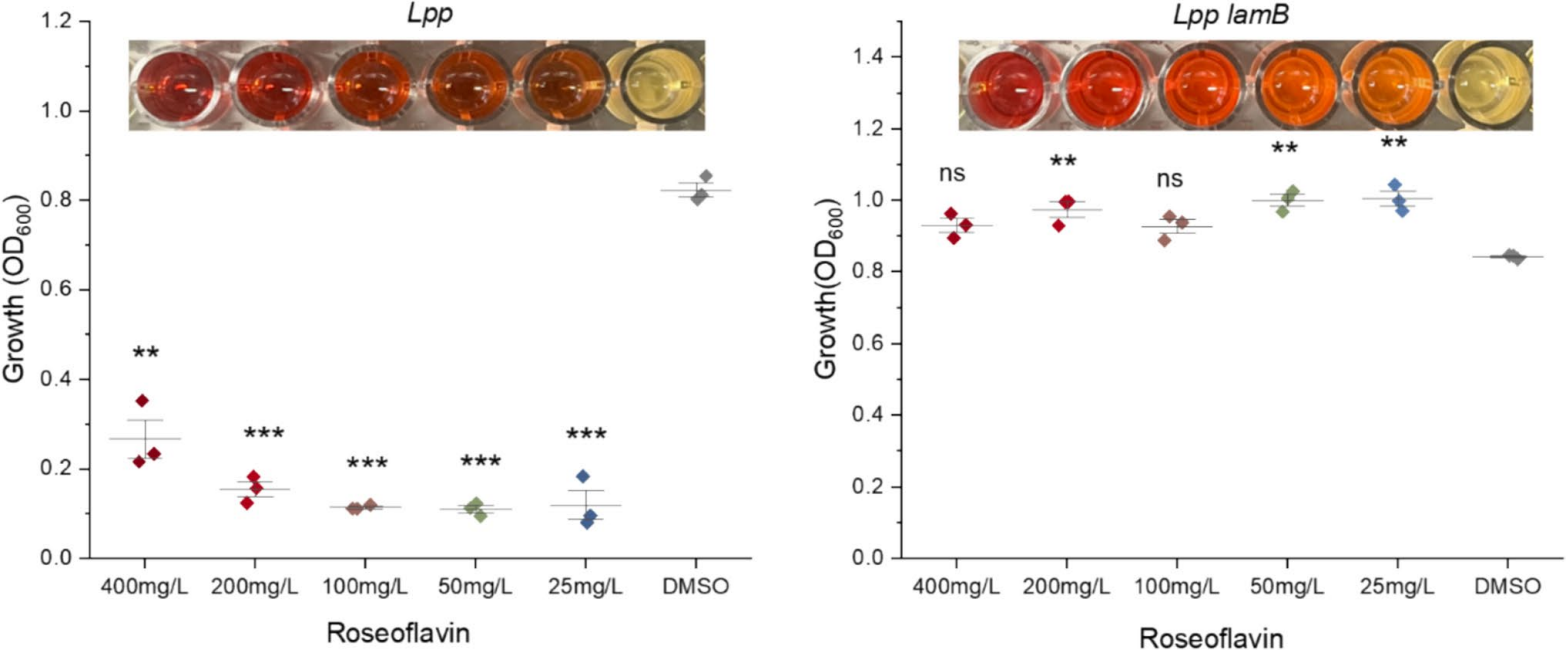
The *L. paraplantarum* CIRM-BIA 1870 *lamB* mutant strain exhibits roseoflavin resistance. *L. paraplantarum* strains were grown in APT media supplemented with varying concentrations of roseoflavin and incubated overnight at 30°C. The *lamB* mutant strain (RNA sequencing suggests it is a riboflavin-overproducing strain) was able to grow normally at high concentrations of roseoflavin. Data represented as mean ± SD of at least three technical replicates from a representative biological replicate. ns (*p* > 0.05) represents no significant difference between test conditions, * (*p* ≤ 0.05), ** (*p* ≤ 0.01), and ***(*p* ≤ 0.001) represent a significant difference between test conditions

To address if riboflavin is overproduced in the *lamB* mutant, we examined how the mutation affected growth in the presence of roseoflavin, a toxic riboflavin analog that disrupts the flavin metabolism [65]. Compared to the wild-type strain, the *lamB* mutant was resistant to roseoflavin, maintaining normal growth even at concentrations of 400 mg/L (Fig. 5). This indicates that the *lamB* mutant is indeed overproducing riboflavin, which likely mitigates roseoflavin toxicity by outcompeting it in metabolic pathways. While roseoflavin resistance and quorum sensing inhibition are distinct phenomena, the observed overproduction of riboflavin in the *lamB* mutant highlights the impact of the *lamBDCA* locus on potential probiotic properties of lactiplantibacilli.

Based on the RNA sequencing data, the insertional inactivation of the *lamB* gene in *L. paraplantarum* indicates that the *lamBDCA* locus impacts global gene expression, affecting various functional categories including stress response, metabolic pathways, and regulatory proteins indicating a broad regulatory role in bacterial gene expression and adaptation.

## Discussion

Anti-virulence therapy aims to attenuate virulence in pathogenic bacteria through virulence-inhibitory compounds or by live bacteria that can modulate virulence gene expression. Here, we present the novel finding that *L. paraplantarum* CIRM-BIA 1870 suppress *S. aureus agr* when grown in co-culture. This is the first time an *agr*-like quorum sensing system has been described for *L. paraplantarum* and that it inhibits *S. aureus agr* quorum sensing. This inhibition likely results from cross-species signaling via the AIP produced by *L. paraplantarum* as the inhibitory effect was significantly reduced by inactivation of the *L. paraplantarum lamBDCA* locus, which is homologous to the *S. aureus agr* system and encodes an AIP with the predicted sequence CTGLF. In contrast, little effect was observed from *L. plantarum* LMG 13556, which produces an AIP identical to that of *L. plantarum* strain WCFS1 (CVGIW) [66].

Previous studies on coagulase-negative staphylococci have shown that spent supernatants can be effectively used to monitor whether a given species produces AIPs that inhibit *S. aureus agr* [16, 56, 57]. However, it has been demonstrated that *L. plantarum*, *L. monocytogenes*, and *C. perfringens* encode an *agr*-like locus and secrete tailless AIPs that undergo an S → N acyl shift in the thiolactone ring at neutral pH, forming homodetic rather than thiolactone AIPs [17]. For *L. monocytogenes* it was, furthermore, shown that the thiolactone AIP most efficiently induces the *L. monocytogenes agr* system compared to the homodetic form [67]. Thus, assuming that the thiolactone form of lactiplantibacilli AIPs inhibits *S. aureus agr,* AIP instability may be a challenge in the cell-free supernatant assay compared to the co-culture conditions where continuous synthesis likely takes place. Differences in the inhibitory potential between the lactiplantibacilli strains may be due to variations in AIP expression levels or in the actual AIP sequence which can impact the rearrangement rate [17]. While the current study relies on supernatants and co-culture conditions, we acknowledge that testing synthetic or purified AIPs would provide clearer insights into their inhibitory activity. Synthetic AIPs will be explored in future studies to further elucidate their potential role in *S. aureus* *agr* inhibition.

When assessing the inhibitory effect of *L. paraplantarum* on *S. aureus agr*, we found that the *lamB* mutant still retained some *agr* inhibitory activity, suggesting additional factors contribute to the observed inhibition. Previously, low pH has been reported to affect *agr* activity [68, 69] while this is not the case for lactate [61]. However, we could exclude this effect in our assays (Figure S3). Also, research has shown that dipeptides produced by *L. reuteri* can inhibit *agr* and reduce virulence factor production in *S. aureus* [4]. Intriguingly, similar dipeptides have been found in *L. plantarum* [62]. We synthesized these dipeptides and observed a minor inhibitory effect against a *S. aureus* strain harbouring the *agrII* system when delivered at relevant concentrations [70]. This suggests that dipeptides may contribute to the inhibition observed in the *lamB* mutant of *L. paraplantarum*.

Several studies have indicated that bacterial biofilm formation is regulated by quorum sensing systems [14, 71]. For *L. plantarum* WCFS1, the Δ*lamA* mutant displayed significantly reduced adherence to glass surfaces compared to the wild-type strain [29]. However, we found no significant differences in biofilm production between *L. paraplantarum* wild-type and *lamB* mutant strain when assessing adherence to plastic and glass surfaces. The absence of similar effects in *L. paraplantarum* CIRM-BIA 1870 could be strain-specific, as the corresponding extracellular polysaccharide synthesis gene clusters cps2 was not differentially expressed in *L. paraplantarum* CIRM-BIA 1870, and our data showed that *L. paraplantarum* CIRM-BIA 1870 generally exhibited lower biofilm formation compared to *L. plantarum* WCFS1. Given the complexity of biofilm formation, other regulatory factors likely play a role in this phenotype. Gene expression analysis revealed that the *lamBDCA* genes promote the expression of stress-related genes, such as stress response genes (e.g., EFP00_RS04570, EFP00_RS08580, EFP00_RS08585, EFP00_RS08595, EFP00_RS08600, and Novel00154), hematin-dependent catalase (EFP00_RS04565, Novel00074), phage holin (EFP00_RS00340), SDR family oxidoreductase (EFP00_RS01460), KUP/HAK/KT family potassium transporter (EFP00_RS08000), hypothetical proteins (EFP00_RS02095, EFP00_RS02100, and EFP00_RS08590), and the ATP-dependent Clp protease subunit ClpL (EFP00_RS04535) consistent with findings from the *ΔlamA* mutant in WCFS1 [30]. In contrast to WCSF1, we observed that in the *L. paraplantarum lamB* mutant, the expression of histidine synthesis and transport genes was increased as were the riboflavin synthesis genes. Histidine transport may be needed for growth at lower pHs [72, 73], while riboflavin synthesis could be important in the human host. Riboflavin (B2 vitamin) is essential for humans and has to be obtained from the diet. Several studies have examined the potential use of lactiplantibacilli as a producer of riboflavin supplements [74, 75]. Our studies show that in *L. paraplantarum* riboflavin synthesis is controlled by *lamBDCA,* and they indicate that repression of the QS system dramatically increases riboflavin synthesis.

Our findings demonstrate that *L. paraplantarum* CIRM-BIA 1870 can inhibit *S. aureus agr*-mediated virulence through its *agr*-like quorum sensing system, highlighting its potential as a probiotic intervention. Similar to AIP variants produced by other *Staphylococcus* species and peptides like fengycin from *B. subtilis*, which reduce *S. aureus* colonization via *agr* repression [15, 16, 19, 20], *L. paraplantarum* could contribute to reducing *S. aureus* prevalence in the gut or nasal cavities. *L. plantarum* is described as a nomadic species found in large variety of habitats but not adapted to any specific environment [76]. *L. paraplantarum* is also found in different habitats [77, 78]. In relation to humans, the former have been isolated from the GI tract and the latter from feces [77]. Thus, their application in the human gastrointestinal tract targeting residing *S. aureus* by AIP-mediated inhibition mechanism may serve as part of a targeted probiotic strategy to naturally reduce pathogenic burden through quorum sensing interference. In this context, it is of interest that Piewngam et al. [19] showed that fengycin-producing *Bacillus* eradicated not only intestinal but also nasal *S. aureus* colonization. Fengycins are chemically unstable and then pose a challenge similar to the lactobacilli AIPs described here, but analogues with enhanced stability have been described [67]. Whether such increased stability would impact the specific mechanisms linking the probiotic effects on nasal and GI-associated *S. aureus* communities presents an interesting topic for future studies.

In conclusion, we report, for the first time, that an AIP-like peptide from *L. paraplantarum* CIRM-BIA 1870 interferes with *S. aureus agr* QS. Notably, live *L. paraplantarum* was needed for effective interference with *S. aureus* virulence in co-culture, underscoring its potential as a probiotic therapy to mitigate virulence. Additionally, our global gene expression analysis revealed that the *lamBDCA* operon has a broad impact on gene expression and controls the synthesis of riboflavin, a metabolite of importance to its probiotic properties. These findings show that *L. paraplantarum* may be a candidate for controlling *S. aureus* virulence and they link the *L. paraplantarum* quorum sensing system to its probiotic properties.

## Supplementary Information

Below is the link to the electronic supplementary material.

Supplementary file1 (PDF 1.95 MB)

## Data Availability

Sequence data that support the findings of this study have been deposited in the National Center for Biotechnology Information (NCBI) with the primary accession code NZ_JBFCVW000000000.1 for *L. paraplantarum* CIRM-BIA 1870, and NZ_JBFCVX000000000.1 for *L. plantarum* LMG 13556.
