# Supplementary material for: Characterization of agr-like Loci in Lactiplantibacillus plantarum and L. paraplantarum and Their Role in Quorum Sensing and Virulence Inhibition of Staphylococcus aureus
Source: Probiotics Antimicrob Proteins. 2025 Feb 19;17(4):2332–47. doi: 10.1007/s12602-025-10476-8 (PMC12405376; doi:10.1007/s12602-025-10476-8)
Supplement: Supplementary file 1 — Supplementary file1 (PDF 1.95 MB) [file 12602_2025_10476_MOESM1_ESM.pdf]

Supporting information

Characterization of *agr*-like loci in *Lactiplantibacillus plantarum* and *L. paraplantarum* and their role in quorum sensing and virulence inhibition of *Staphylococcus aureus*

Weizhe Wang[a], Ifigeneia Kyrkou[a], Martin S. Bojer[a], Dina Kalloubi[b], Abdul Jabbar Kali[b], Miguel Alena-Rodriguez [b], Jørgen J. Leisner[a], Stephanie Fulaz[a,]*, Hanne Ingmer[a,]*

[a]Department of Veterinary and Animal Sciences, University of Copenhagen

[b]Center for Biopharmaceuticals & Department of Drug Design and Pharmacology, Faculty of Health and Medical Sciences, University of Copenhagen, Jagtvej 160, DK-2100, Copenhagen, Denmark.

* Corresponding authors: hi@sund.ku.dk

stephanie.silva@sund.ku.dk

Table of contents

Supplementary Figures………3

Fig. S1. The Growth curve and YFP accumulation of *L. plantarum* LMG 13556 wild-type and *lam*B mutant strains inhibiting *S. aureus* RNAIII expression by co-culturing…………………………..3

Fig. S2. The Growth curve and YFP accumulation of *L. paraplantarum* CIRM-BIA 1870 wild-type and *lam*B mutant strains inhibiting *S. aureus* RNAIII expression by co-culturing…………..4

Fig. S3. The Growth curve and YFP accumulation of *S. aureus* in different pH media…………..5

Fig. S4. The *L. paraplantarum* CIRM-BIA 1870 wild-type and *lam*B mutant strains exhibit no hemolysis………...6

Fig. S5. (A) Correlation of gene expression between three independent biological replicates is shown by the Pearson correlation coefficient……………………………………………………………6

Supplementary tables………7

Table S1. Information on LamBDCA and LamKR protein sequences used in this study, as retrieved from the NCBI RefSeq database…………………………………………………………………………7

Table S2. Structure of dipeptides produced by Lactiplantibacilli…………………………………………8

Table S3. Differentially expressed genes between *L. paraplantarum lamB* mutant and the wild-type strain (|Log2Fold Change|>1.5)……………………………………………………………………………9

References……13

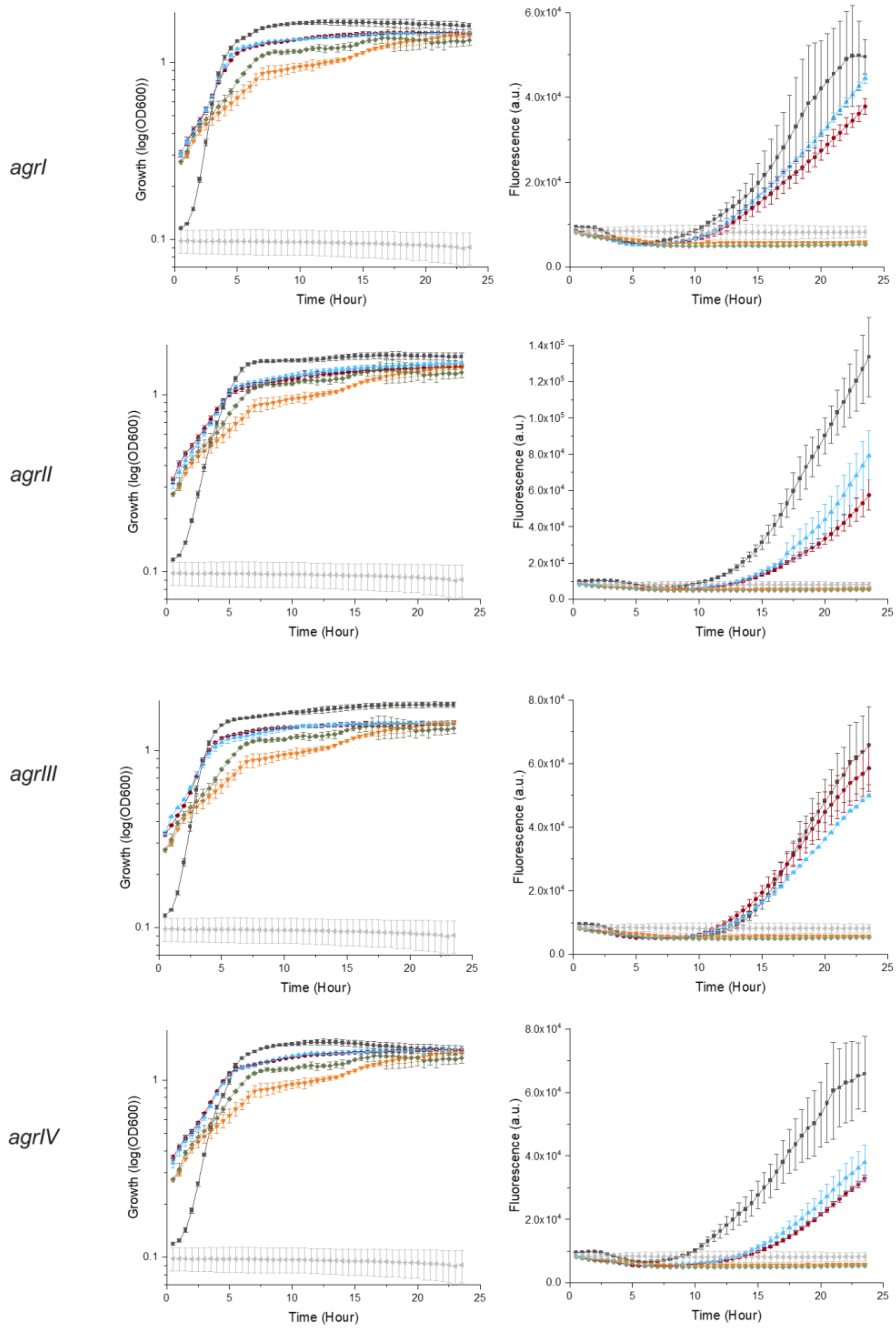

Fig. S1. The Growth curve and YFP accumulation of *L. plantarum* LMG 13556 wild-type and *lam*B mutant strains inhibiting *S. aureus* RNAIII expression by co-culturing. Activity of four *S. aureus agr* group reporters assessed by RNAIII expression (P3-yfp) measured as fluorescence, either alone or in co-culture with wild-type or *lamB* mutant strains. The OD_{600} and YFP fluorescence (λexc 500 nm, λem 541 nm) were measured every 30min over 24 hours. , *S. aureus agrI-IV* YFP reporters; , YFP reporters co-culturing with *L. plantarum* LMG 13556; , YFP reporters co-culturing with *L. plantarum* LMG 13556 *lamB;* , *L. plantarum* LMG 13556; , *L. plantarum* LMG 13556 *lamB;* , Mix-media.

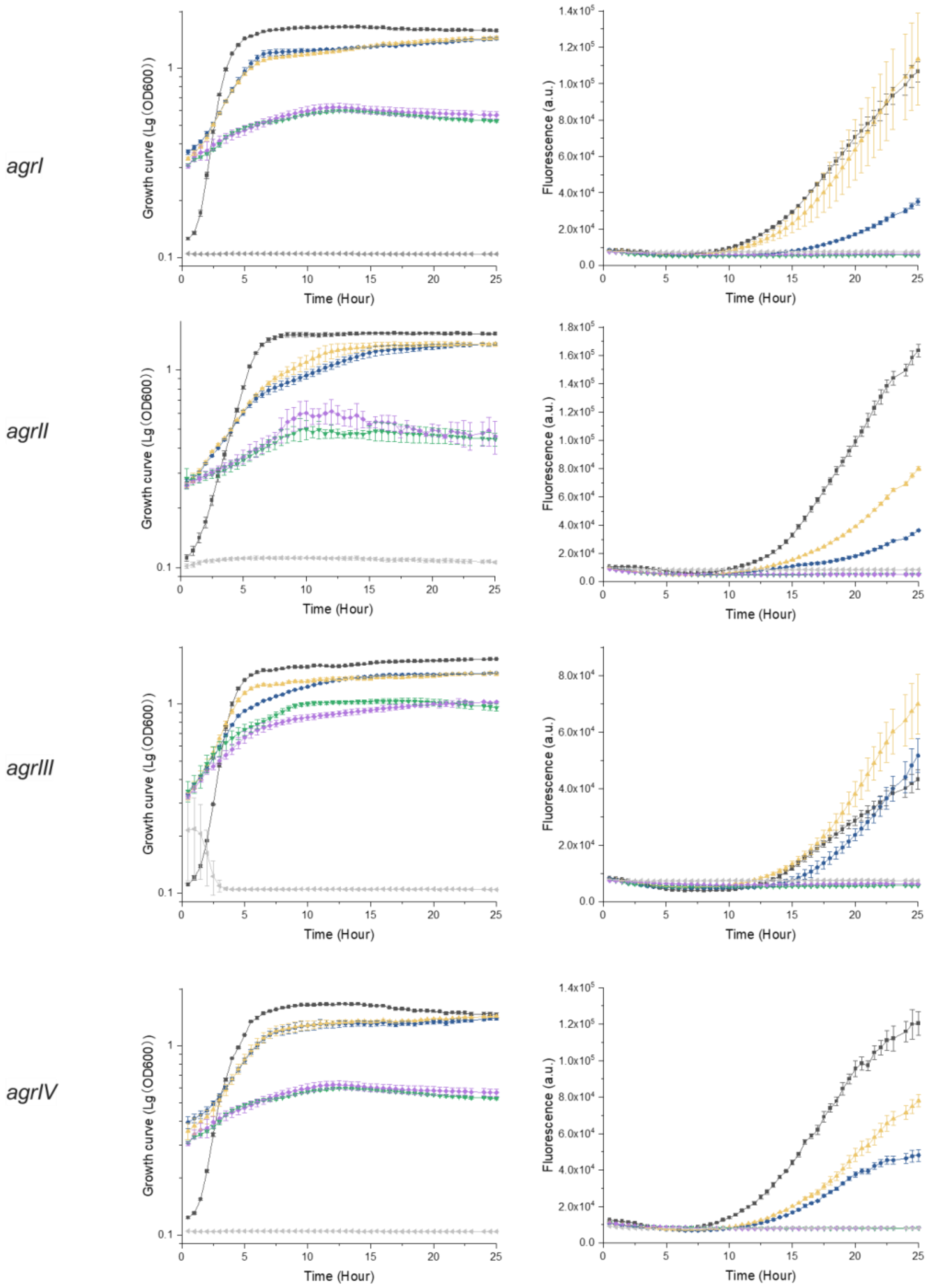

Fig. S2. The Growth curve and YFP accumulation of *L. paraplantarum* CIRM-BIA 1870 wild-type and *lam*B mutant strains inhibiting *S. aureus* RNAIII expression by co-culturing. Activity of four *S. aureus agr* group reporters assessed by RNAIII expression (P3-yfp) measured as fluorescence, either alone or in co-culture with wild-type or *lamB* mutant strains. The OD_{600} and YFP fluorescence (λexc 500 nm, λem 541 nm) were measured every 30min over 24 hours. , *S. aureus agrI-IV* YFP reporters; , YFP reporters co-culturing with *L. paraplantarum* CIRM-BIA 1870; , YFP reporters co-culturing with *L. paraplantarum* CIRM-BIA 1870 *lamB;* , *L. paraplantarum* CIRM-BIA 1870; , *L. paraplantarum* CIRM-BIA 1870 *lamB;* , Mix-media.

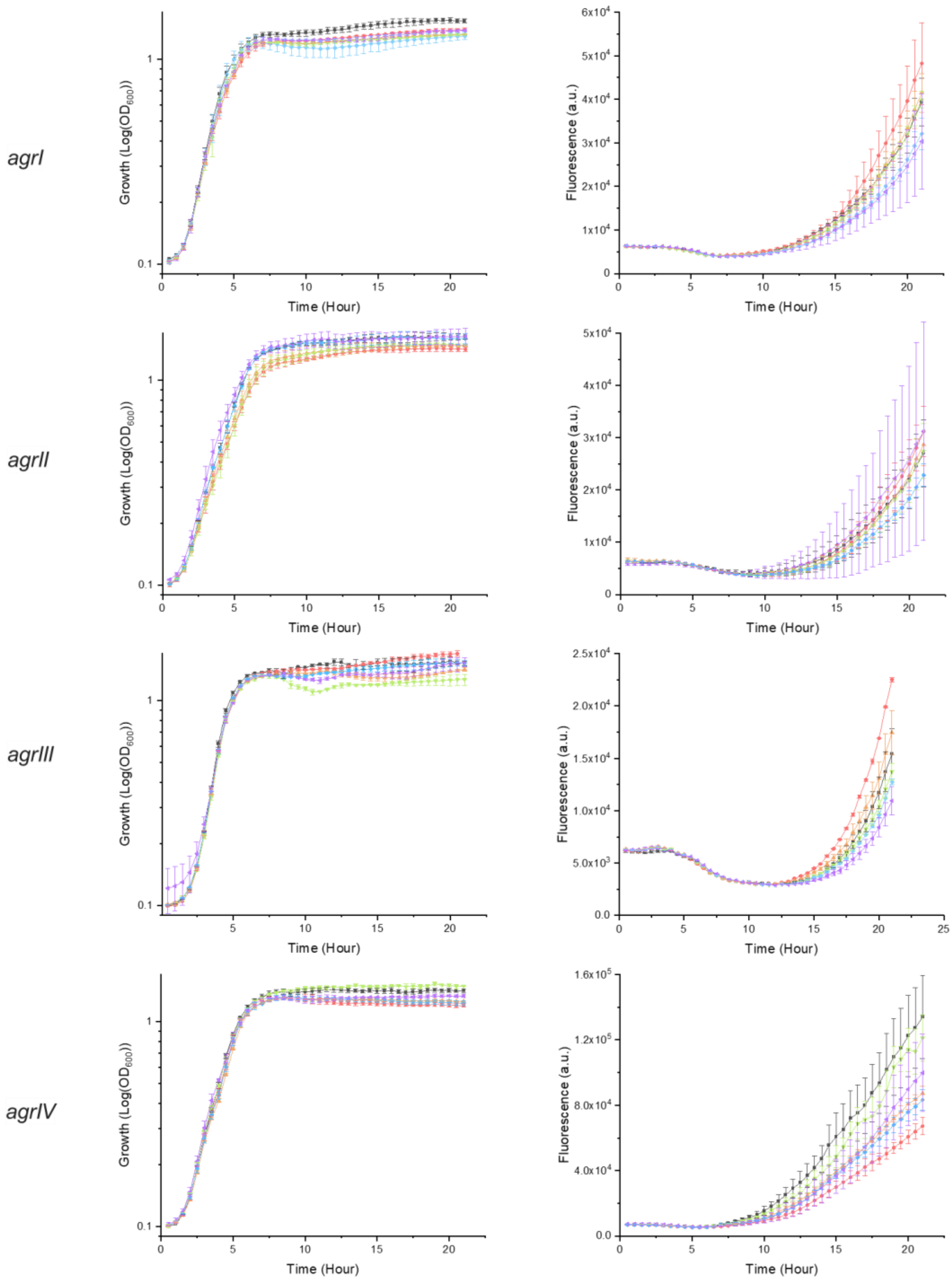

Fig. S3. The Growth curve and YFP accumulation of *S. aureus* in different pH media. Twenty-five microliters of different pH TSB was mixed with 75 µL of the *S. aureus* reporter strain in a 96-well black plate (Fisherbrand™), with TSB added to reach a final volume of 150 µL. Activity of four *S. aureus agr* group reporters assessed by RNAIII expression (P3-yfp) measured as fluorescence, with the addition of pH-adjusted TSB. The OD_{600} and YFP fluorescence (λexc 500 nm, λem 541 nm) were measured every 30min over 24 hours. ▪, *S. aureus agrI-IV* YFP reporters; ●, pH 4.5; ▲, pH 5.0; ▼, pH 5.5; ◆, pH 6.0; ◀, pH 6.5.

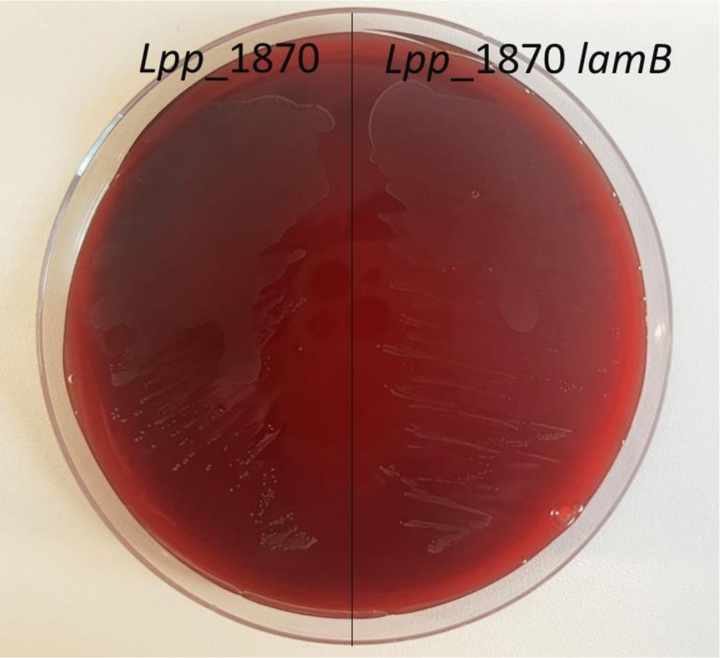

Fig. S4. The *L. paraplantarum* CIRM-BIA 1870 wild-type and *lam*B mutant strains exhibit no hemolysis. *L. paraplantarum* strains were streaked on APT agar supplemented with 5% cow blood and incubated overnight at 30°C, showing no visible hemolytic zones.

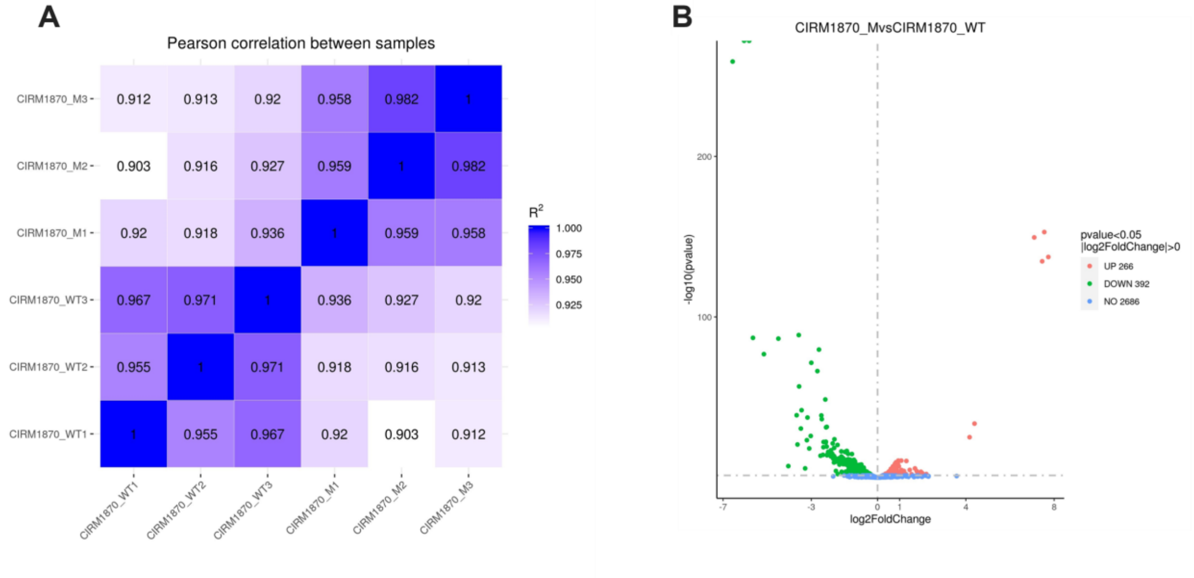

Fig. S5. (A) Correlation of gene expression between three independent biological replicates is shown by the Pearson correlation coefficient. (B) A total of 658 genes were found to be differentially expressed, with 266 genes up-regulated and 392 genes down-regulated in the *L. paraplantarum lamB* mutant compared to the wild-type.

Table S1. Information on LamBDCA and LamKR protein sequences used in this study, as retrieved from the NCBI RefSeq database. The first literature reference to present each of these proteins is also disclosed.

Strain (RefSeq)	Protein	NCBI Protein SeqID	NCBI Record Annotation	Reference
L. plantarum LMG 13556 (NZ_JBFCVX000000000.1)	LamB	WP_053567040.1	accessory gene regulator AgrB	This study
	LamD	WP_011102233.1	cyclic lactone autoinducer peptide	This study
	LamC	WP_011102232.1	GHKL domain-containing protein	This study
	LamA	WP_015826152.1	LytTR family DNA-binding domain-containing protein	This study
	LamK	WP_003642334.1	GHKL domain-containing protein	This study
	LamR	WP_011102036.1	LytTR family DNA-binding domain-containing protein	This study
L. paraplantarum CIRM-BIA 1870 (NZ_JBFCVW000000000.1)	LamB	WP_033611555.1	accessory gene regulator AgrB	This study
	LamD	WP_131510691.1	cyclic lactone autoinducer peptide	This study
	LamC	WP_260352128.1	GHKL domain-containing protein	This study
	LamA	WP_033611558.1	LytTR family DNA-binding domain-containing protein	This study
	LamK	WP_260351803.1	GHKL domain-containing protein	This study
	LamR	WP_260351802.1	LytTR family DNA-binding domain-containing protein	This study
L. paraplantarum WCFS1 (NC_004567.2)	LamB	WP_011102234.1	accessory gene regulator AgrB	[1]
	LamD	WP_011102233.1	cyclic lactone autoinducer peptide	[1]
	LamC	WP_011102232.1	GHKL domain-containing protein	[1]
	LamA	WP_011102231.1	LytTR family DNA-binding domain-containing protein	[1]
	LamK	WP_003642334.1	GHKL domain-containing protein	[2]
	LamR	WP_011102036.1	LytTR family DNA-binding domain-containing protein	[2]
S. aureus subsp. aureus DSM 20231 (NZ_CP104478.1)	AgrB	WP_001105707.1	accessory gene regulator AgrB	[3]
	AgrD	WP_001093929.1	cyclic lactone autoinducer peptide AgrD	[3]
	AgrC	WP_001554031.1	quorum-sensing sensor histidine kinase AgrC	[3]
	AgrA	WP_001836294.1	quorum-sensing response regulator AgrA	[3]

Table S2. Structure of dipeptides produced by Lactiplantibacilli

ID	Depetide	Structure
DK1	Cyclo(L-Phe-L-Pro)	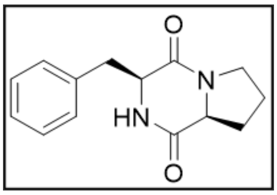
DK2	Cyclo(L-Tyr-L-Pro)	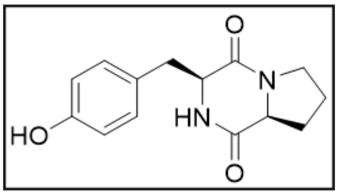

Table S3. Differentially expressed genes between *L. paraplantarum lamB* mutant and the wild-type strain (|Log2Fold Change|>1.5).

Gene_ID	Log2Fold Change	Description
EFP00_RS00340	-2.3	phage holin
EFP00_RS00345	-2.4	hypothetical phage protein
EFP00_RS00350	-2.3	phage endolysin
EFP00_RS00355	-2.1	hypothetical phage protein
EFP00_RS00360	-2.3	hypothetical phage protein
EFP00_RS00365	-2.2	phage tail protein
EFP00_RS00370	-2.1	phage tail protein
EFP00_RS00375	-2.1	phage tail protein
EFP00_RS00380	-1.9	hypothetical phage protein
EFP00_RS00385	-2.0	DUF6096 family protein
EFP00_RS00390	-2.1	phage major tail protein
EFP00_RS00395	-2.1	hypothetical protein
EFP00_RS00400	-2.2	phage tail protein
EFP00_RS00405	-2.1	phage structural protein
EFP00_RS00410	-2.1	phage head-tail connector protein
EFP00_RS00415	-2.1	hypothetical phage protein
EFP00_RS00420	-2.0	major capsid protein
EFP00_RS00425	-2.0	hypothetical phage protein
EFP00_RS00430	-1.9	DUF4355 domain-containing protein
EFP00_RS00435	-1.9	hypothetical phage protein
EFP00_RS00445	-1.6	minor capsid protein
EFP00_RS00450	-1.7	ribosomal-processing cysteine protease Prp
EFP00_RS00455	-1.8	phage portal protein

Gene_ID	Log2Fold Change	Description
EFP00_RS00460	-1.7	phage terminase large subunit
EFP00_RS00465	-1.7	phage terminase small subunit
EFP00_RS00470	-1.6	DUF6275 family protein
EFP00_RS01460	-2.7	SDR family oxidoreductase
EFP00_RS01800	-1.6	DNA starvation/stationary phase protection protein
EFP00_RS02095	-2.5	hypothetical protein
EFP00_RS02100	-2.6	hypothetical phage protein
EFP00_RS02715	-2.0	zinc-binding dehydrogenase
EFP00_RS04220	-2.3	pyridoxamine 5'-phosphate oxidase family protein
EFP00_RS04530	-6.0	hypothetical protein
EFP00_RS04535	-3.0	ATP-dependent Clp protease ATP-binding subunit ClpL
EFP00_RS04540	-3.6	homologue of accessory gene regulator protein B, LamB
EFP00_RS04545	-5.6	Cyclic lactone autoinducer peptide precursor, LamD
EFP00_RS04550	-5.8	homologue of histidine kinase sensor protein C, LamC
EFP00_RS04555	-5.8	homologue of accessory gene Response Regulator protein A, LamA
EFP00_RS04560	-6.6	Spx/MgsR family RNA polymerase-binding regulatory protein
EFP00_RS04565	-1.9	catalase
EFP00_RS04570	-2.3	GlsB/YeaQ/YmgE family stress response membrane protein
EFP00_RS04575	-2.0	hypothetical protein
EFP00_RS04590	-1.8	NAD(P)/FAD-dependent oxidoreductase
EFP00_RS04615	-3.6	Spx/MgsR family RNA polymerase-binding regulatory protein
EFP00_RS05135	-2.0	glutamate decarboxylase
EFP00_RS05840	-1.6	ABC-transporter ATP-binding protein

Gene_ID	Log2Fold Change	Description
EFP00_RS05850	-1.7	glycerol kinase GlpK
EFP00_RS05855	-1.9	type 1 glycerol-3-phosphate oxidase
EFP00_RS07950	-1.6	DNA starvation/stationary phase protection protein
EFP00_RS08000	-3.0	KUP/HAK/KT family potassium transporter
EFP00_RS08580	-3.6	GlsB/YeaQ/YmgE family stress response membrane protein
EFP00_RS08585	-3.5	alkaline shock response membrane anchor protein, AmaP
EFP00_RS08590	-3.2	hypothetical protein
EFP00_RS08595	-3.7	alkaline shock protein 23/Gls24 family envelope stress response protein
EFP00_RS08600	-3.4	alkaline shock protein /Gls24 family envelope stress response protein
EFP00_RS09260	-1.6	iron ABC transporter permease
EFP00_RS09265	-3.2	universal stress protein
EFP00_RS09415	-2.7	CsbD family protein
EFP00_RS09420	-2.4	integral membrane protein
EFP00_RS09425	-2.5	integral membrane protein
EFP00_RS09430	-2.3	diacylglycerol kinase family protein
EFP00_RS09450	-2.0	hypothetical protein
EFP00_RS10185	-1.5	hypothetical protein
EFP00_RS11495	-1.8	PepSY domain-containing protein
EFP00_RS12620	-1.5	hypothetical protein
Novel00072	-3.1	Histidine kinase with LytTr DNA-binding domain
Novel00073	-1.6	ArsC family
Novel00074	-4.5	Catalase
Novel00154	-2.5	alkaline shock protein 23
Novel00170	-4.0	Universal stress protein family

Gene_ID	Log2Fold Change	Description
EFP00_RS00845	7.6	5-amino-6-(5-phosphoribosylamino) uracil reductase RibD
EFP00_RS00850	7.7	riboflavin synthase, RibB
EFP00_RS00855	7.5	riboflavin biosynthesis protein, RibA
EFP00_RS00860	7.1	6,7-dimethyl-8-ribityllumazine synthase, RibH
EFP00_RS00870	4.4	NAD(P)H-binding protein
EFP00_RS00875	4.2	NAD(P)H-binding protein
EFP00_RS04135	1.7	phage major capsid protein
EFP00_RS04145	1.8	phage terminase large subunit
EFP00_RS04150	1.8	phage terminase small subunit
EFP00_RS04190	2.2	hypothetical protein
EFP00_RS07265	2.1	tRNA-Thr
EFP00_RS11345	1.8	BspA family leucine-rich repeat surface protein
EFP00_RS14135	2.0	imidazoleglycerol-phosphate dehydratase, HisB
EFP00_RS14140	2.0	imidazole glycerol phosphate synthase subunit, HisH
EFP00_RS14145	1.5	1-(5-phosphoribosyl)-5-[(5-phosphoribosylamino) methylideneamino] imidazole-4-carboxamide isomerase
EFP00_RS14825	2.2	tRNA-Asp
Novel00009	1.9	Permease family

References

1. Sturme, M.H.J., et al., *An agr-Like Two-Component Regulatory System in Lactobacillus plantarum Is Involved in Production of a Novel Cyclic Peptide and Regulation of Adherence.* Journal of Bacteriology, 2005. **187**(15): p. 5224-5235.DOI: 10.1128/Jb.187.15.5224-5235.2005.
2. Fujii, T., et al., *Two Homologous Agr-Like Quorum-Sensing Systems Cooperatively Control Adherence, Cell Morphology, and Cell Viability Properties in WCFS1.* Journal of Bacteriology, 2008. **190**(23): p. 7655-7665.DOI: 10.1128/Jb.01489-07.
3. Wollesen, M., et al., *Polyether Ionophore Antibiotics Target Drug-Resistant Clinical solates, Persister Cells, and Biofilms.* Microbiol Spectr, 2023. **11**(4): p. e0062523.DOI: 10.1128/spectrum.00625-23.
